# The role of the lysine histone methylase KMT2D in chronic myeloid leukemia

**DOI:** 10.3389/fphar.2025.1652373

**Published:** 2025-09-16

**Authors:** Lina Schlemminger, Inga Nagel, Inga Vater, Ingolf Cascorbi, Meike Kaehler

**Affiliations:** ^1^ Institute of Experimental and Clinical Pharmacology, University Hospital Schleswig-Holstein, Campus Kiel, Kiel, Germany; ^2^ Institute of Human Genetics, University Hospital Schleswig-Holstein, Campus Kiel, Kiel, Germany

**Keywords:** chronic myeloid leukemia, drug resistance, imatinib, KMT2D, epigenetics, histone modification

## Abstract

Chronic myeloid leukemia (CML) can be effectively treated inhibiting the disease-causing BCR::ABL1 kinase by tyrosine kinase inhibitors (TKIs). Although therapy is initially tremendously successful, resistance may occur in up to 25% of CML patients. Besides aberrations in the BCR::ABL1 kinase domain, a variety of resistance mechanisms are currently discussed, among them epigenetic reprogramming. The histone-modifying enzyme lysine methyltransferase 2D (KMT2D/MLL2) belongs to the most frequently mutated genes in cancer and is also known for its association with hereditary Kabuki syndrome. However, its role in CML is widely unknown. In the present study, we analyzed the role of the *KMT2D* p. (Arg191Trp) variant in imatinib-resistant CML, which was recurrently acquired in imatinib resistance *in vitro*. SiRNA-mediated *KMT2D* knockdown, but also introduction of the p. (Arg191Trp) variant into treatment-naïve K-562 cells led to impaired imatinib susceptibility visible by increased cell numbers, proliferation rates and metabolic activities under imatinib exposure (p < 0.001). The effect of *KMT2D* p. (Arg191Trp) could be overcome by inhibiting histone demethylation with the demethylase inhibitor LSD1. In addition, rescue of *KMT2D* expression in imatinib-resistant cells reinstated the response to imatinib treatment. Furthermore, gene expression analysis revealed upregulation of *CCNE2* in cells harboring *KMT2D* p. (Arg191Trp) potentially explaining increase in cell proliferation under imatinib exposure. Overall, our findings demonstrate that the loss of the tumor suppressor *KMT2D* promotes TKI resistance in CML. Thus, *KMT2D* status could serve as an additional biomarker for TKI resistance, while restoration of its expression might be a therapeutic option to overcome this resistance.

## Graphical Abstract

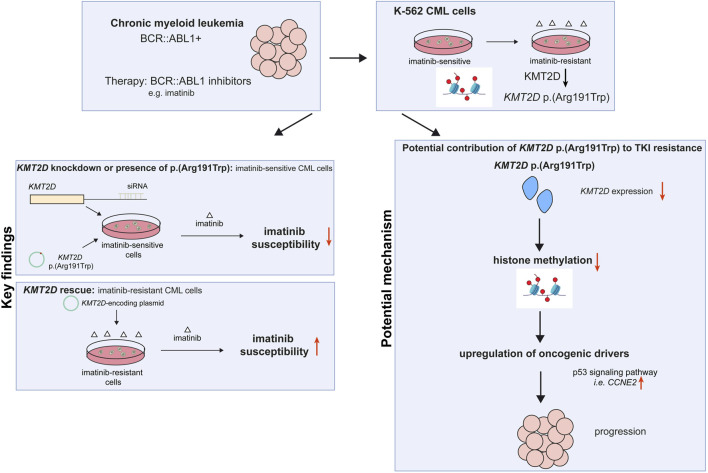

## 1 Introduction

Chronic myeloid leukemia (CML) is a rare hematopoietic neoplasm predominantly caused by reciprocal translocation t (9; 22) (q34; q11), resulting in the formation of the BCR::ABL1 fusion gene, which is considered as the hallmark of the disease (Nowell and Hungerford, 1960; Rowley, 1973). Since the development of tyrosine kinase inhibitors (TKIs), which inhibit the disease-causing BCR::ABL1 kinase and prevent downstream target phosphorylation, CML can be effectively treated (Druker et al., 1996). With an overall survival rate of 83%, the use of TKIs in CML became a role model for successful targeted therapy regimens (Hochhaus et al., 2017).

Nevertheless, up to 25% of patients undergoing TKI therapy suffer from TKI failure due to the development of TKI resistances within 5 years after therapy onset (Milojkovic and Apperley, 2009). Besides mutations in the BCR::ABL1 kinase, in particular in the kinase domain, TKI resistance can be caused by alternative signaling pathway activation, persistent leukemic stem cells or drug transporters (Bixby and Talpaz, 2011; Kaehler and Cascorbi, 2023). In addition, secondary driver gene mutations or epigenetic factors might lead to disease progression and/or drug resistance (Minciacchi et al., 2021).

There is increasing evidence that epigenetic modifiers play a role in TKI-resistant CML. For instance, inhibitors of histone deacetylases (HDAC), an enzyme class responsible for the removal of acetyl groups from histones, were considered to eradicate CML leukemic stem cells (Minciacchi et al., 2021). In addition, differences in the methylation pattern and the expression of lysine methyltransferases, e.g. *EHMT1* or *EHMT2*, were observed in CML (Loh et al., 2014). Besides, histone-modifiers of the KMT2 (histone-lysine N-methyltransferase 2) family are also frequently associated with the development of cancer, in particular KMT2A/MML1 (mixed lineage leukemia 1), which dearrangement leads to an oncogenic fusion protein in acute lymphoblastic leukemia (Ford and Dingwall, 2015).

Within this KMT2 family, the mixed-lineage leukemia 2/histone lysine methyltransferase 2D (*MLL2*/*KMT2D*) gene encodes a large 5,537 aa protein involved in mono-methylation of histone H3K4, especially in enhancer regions, thereby being involved in transcriptional activation (Rao and Dou, 2015). In Kabuki syndrome, a rare developmental disorder with craniofacial malfunctions, growth delay, impaired immune system, kidney and heart function (Boniel et al., 2024), germline missense mutations in *KMT2D* can be detected in 56%–75% of cases (Bogershausen and Wollnik, 2013). Regarding somatic mutations, *KMT2* genes, especially *KMT2C* and *KMT2D*, were found to be among the most frequently mutated genes in cancer (Kandoth et al., 2013). Nonetheless, the role of *KMT2D* in CML is still widely unknown.

In an in vitro-cell line model of TKI resistance, we detected the recurrent *KMT2D* variant c.571C>T, p. (Arg191Trp) in imatinib-resistant cells by exome sequencing (Kaehler et al., 2023). This raised the question on the role of *KMT2D* and the effect of the observed *KMT2D* variant in imatinib resistant CML. Here, we analyzed the role of *KMT2D* and the *KMT2D* variant p. (Arg191Trp) and further epigenetic modifiers using an in vitro-imatinib resistance model providing new insights into the role of *KMT2D* in TKI-resistant CML.

## 2 Materials and methods

### 2.1 Reagents, cell lines, and generation of resistant cells

Cell experiments were performed using K-562 cells (RRID: CVCL_0004), a cell line derived from a 53-year-old female CML patient in blast crisis (Lozzio and Lozzio, 1975) provided by the German Collection of Microorganisms and Cell Cultures (DSMZ, Braunschweig, Germany). Cells were maintained as previously described (Turrini et al., 2012; Kaehler et al., 2022). Imatinib-resistant replicates were obtained by exposing treatment-naïve K-562 cells to increasing concentrations of imatinib, resulting in cells resistant to 0.5 µM and 2 µM imatinib.

### 2.2 RNA and DNA extraction

RNA extraction was performed using E. Z.N.A total RNA Kit I (Omega bio-tek, Norcross, Georgia, United States) following the manufacturer’s instructions with the added step of centrifuging the cell lysate within QIAshredder homogenizers (Qiagen, Hilden, Germany) for 1 min at 10,000 x g after exposure to the lysis buffer to enhance RNA extraction. DNA extraction was performed using the Gentra Puregene Kit (Qiagen).

### 2.3 Reverse transcription quantitative polymerase chain reaction (RT-qPCR)

Reverse transcription of 1 µg RNA was conducted with the High-Capacity cDNA Reverse Transcription Kit (Thermo Fisher Scientific, Darmstadt, Germany) according to the manufacturer’s protocol. RT-qPCR was performed with the QuantStudio 7 Flex (Thermo Fisher Scientific) applying default cycling conditions. Samples were examined in triplicates using the TaqMan Universal Master Mix without UNG (Thermo Fisher Scientific) and the following TaqMan assays obtained from Thermo Fisher Scientific: *KMT2D* (Hs00912419_m1), *CDK4* (Hs00364847_m1), *CCND3* (Hs00236949_m1), *CCNE2* (Hs00180319_m1), *TBP* (Hs00427620_m1), *GAPDH* (Hs02786624_g1), *18S* (Hs99999901_s1). The cycle threshold (CT) value of the target genes were normalized to the housekeeping genes *TBP*, *GAPDH* and *18S* with relative mRNA expression being calculated as 2^−ΔΔCT^ (Livak and Schmittgen, 2001).

### 2.4 In-depth-sequencing

Amplicons of the *KMT2D* gene were generated using the AmpliTaq Gold 360 Mastermix (Thermo Fisher Scientific) and the primers 5′-GAT​GTC​CAT​GGC​TTT​ACC​ACT​TCC​CCT​GC-3′ and 5′-AAA​GCC​ATG​GAC​ATC​CAG​GTG​AGC​GG-3’ (obtained from Merck, Darmstadt, Germany) with an annealing temperature of 58 °C and an elongation time of 7 min. The PCR products were purified using the GeneJET Gel extraction Kit (Thermo Fisher Scientific). Next-Generation Sequencing was performed using the Nextera XT Sequencing kit (Illumina, San Diego, California, United States) adhering to the manufacturer’s protocol as previously described (Kaehler et al., 2021; Kaehler et al., 2023).

### 2.5 Cloning and plasmids

The *KMT2D*-encoding plasmid was provided by Promega (Cat# FHC12732, Madison, Wisconsin, United States) and the plasmid harboring the *KMT2D* p. (Trp191Arg) variant was obtained by mutagenesis at GenScript (Rijswijk, Netherlands). The empty pFN21A vector was obtained through restriction enzyme cloning with AsiSI and Pme1 (both New England Biolabs). Plasmid DNA was isolated using PureYield Plasmid Multiprep System (Promega) or NucleoBond Xtra Midi Kit (Macherey Nagel GmbH).

### 2.6 Transient transfection

Transient transfection was performed with 4 x 10^6^ cells using the Amaxa Cell Line Nucleofector Kit V (Lonza, Basel, Switzerland) with the Nucleofector I device (Lonza) following the manufacturer’s recommendation for K-562 cells. After respective incubation periods, cell seeding was carried out to investigate cell viability under exposure to 2 µM imatinib in cellular fitness assays as described below. For the *KMT2D* knockdown, treatment-naïve cells were transfected with 200 nM Ambion MLL2 Silencer siRNA (Cat# AM51331) or negative control #1 siRNA (Cat# AM4611) with subsequent cell seeding after an incubation period of 6 h. Imatinib-resistant cells were transfected with 10 µg of a *KMT2D*-encoding plasmid or empty pFN21A vector followed by cell seeding after 1 h incubation. Furthermore, treatment-naïve K-562 cells were transfected with 10 µg of a plasmid harboring the *KMT2D* p. (Trp191Arg) variant, *KMT2D* wild-type or the empty pFN21A vector as negative control. Cells were seeded 24 h after transfection. The cells were additionally exposed to 100 µM LSD1 inhibitor or DMSO as a solvent control.

### 2.7 Cellular fitness assays

Cells were seeded into 12-well plates with 1 x 10^6^ cells/mL for the Ki-67 assay as well as immunoblotting, whereas 96-well plates were used to determine cell numbers with 2 x 10^5^ cells/200 µL medium and metabolic activity with 5 x 10^4^ cells/100 µL medium. Cells were exposed to either 2 µM imatinib or medium and incubated at 37 °C. To determine cell numbers, the cell suspension was mixed with trypan blue (Sigma Aldrich) to mark viable, unstained cells, which were then quantified with a Fuchs-Rosenthal cell counting chamber after 24 and 48 h. Metabolic activity was measured using the WST assay (Merck) as previously described (Kaehler et al., 2017).

Cell proliferation was determined 24 h after transient transfection using the Human Antigen Ki-67 ELISA Kit (Cat# MBS764543, MyBioSource, San Diego, California, United States) with 10 µg of protein according to the manufacturer’s protocol. To determine the influence of imatinib on cell viability, the results of cells treated with imatinib were normalized to treatment-naïve cells.

### 2.8 Whole-cell lysates and immunoblotting

Cell lysis and immunoblots were performed as described elsewhere (Kaehler et al., 2017; Waetzig et al., 2019; Bruhn et al., 2020). Using 15% v/v polyacrylamide gels, 20 µg of protein were transferred onto nitrocellulose membranes and membranes were probed with the following antibodies: Histon H3: Cat# sc-517576 (Santa Cruz, Dallas, Texas, United States), RRID: AB_2848194, 1:250; H3K4me1: Cat# 710795-20UG (Thermo Fisher Scientific), RRID: AB_2848515, 1:1,000; HSP90: Cat# 4877 (Cell Signaling Technology, Danvers, Massachusetts, United States), RRID: AB_2233307, 1:1,000; anti-mouse: Cat# 926-68070, RRID: AB_10956588, Cat# 926-32210, RRID: AB_621842; anti-rabbit: Cat# 926-68071, RRID: AB_10956166, Cat# 926-32211, RRID: AB_621843; all 1:10,000, LiCOR (Bad Homburg, Germany). Primary antibodies were diluted with the Intercept TBS Blocking Buffer supplemented with 0.2% v/v Tween20, whereas secondary antibodies were diluted in TBS with 0.1% v/v Tween20.

### 2.9 Inhibition assays

Inhibition experiments were conducted in 96-well plates with triplicates of 5 x 10^4^ cells/100 µL medium supplemented with 2 µM imatinib with DMSO as solvent control. LSD1 was inhibited using 1–200 µM LSD1 Inhibitor II (S2101, Merck Millipore, United States). Furthermore, 1–100 µM of the histone-deacetylase (HDAC) inhibitor vorinostat (Cat# SML0061, Merck) and 0.1–250 µM of the DNA-methyltransferase (DNMT) inhibitor 5′-azacytidine (Hölzel Diagnostika, Köln, Germany) were used. After an incubation of 48 h at 37 °C, metabolic activity was measured as described above. IC50 values were calculated by non-linear regression with variable slope (four parameters) for N = 3 including at least six concentrations.

### 2.10 Meta-analyses of exome sequencing and genome-wide gene expression data

Exome sequencing data of TKI-resistant biological replicate cell lines was obtained from the European Nucleotide Archive (ENA), accession number PRJEB60565. *KMT2D* variants were identified as previously described (Kaehler et al., 2023). In silico prediction of the variant effect was performed using gnomAD (gnomad.broadinstitute.org). Genome-wide gene expression data was derived from the GEO datasets GSE227347 and GSE203342 as previously published (Kaehler et al., 2022; Kaehler et al., 2023). Comparing treatment-naïve and imatinib-resistant cell lines, genes with a fold change ±2 and a false discovery rate (FDR)-corrected p-value p < 0.05 were considered to be differentially expressed. Venn diagrams for the comparison of these differentially expressed genes with the *KMT2D* essentiality network (Takemon et al., 2024) were obtained using the PNNL software (omics.pnl.gov (Oliveros, 2007)). KEGG pathway prediction was performed using DAVID Functional Annotation Tool (DAVID Bioinformatics Resources (Huang da et al., 2009; Sherman et al., 2022)) and interaction networks using the STRING database (string-db.org. Version 12.0 with medium confidence).

### 2.11 Software and statistical analysis

Primers were designed with the NCBI primer design tool (National Center for Biotechnology Information, Bethesda, Maryland, United States). Unless indicated otherwise, statistical analyses were performed using student’s t-tests or One-way ANOVA followed by Dunnett’s tests to examine multiple comparisons with the GraphPad Prism software (Version 10.2.3 for Windows, San Diego California, United States). For all experiments, data from at least three replicates was analyzed. Results were considered as statistically significant with a p-value <0.05.

## 3 Results

### 3.1 *KMT2D* expression and presence of p.Arg191Trp in imatinib-resistant CML

**FIGURE 1:**
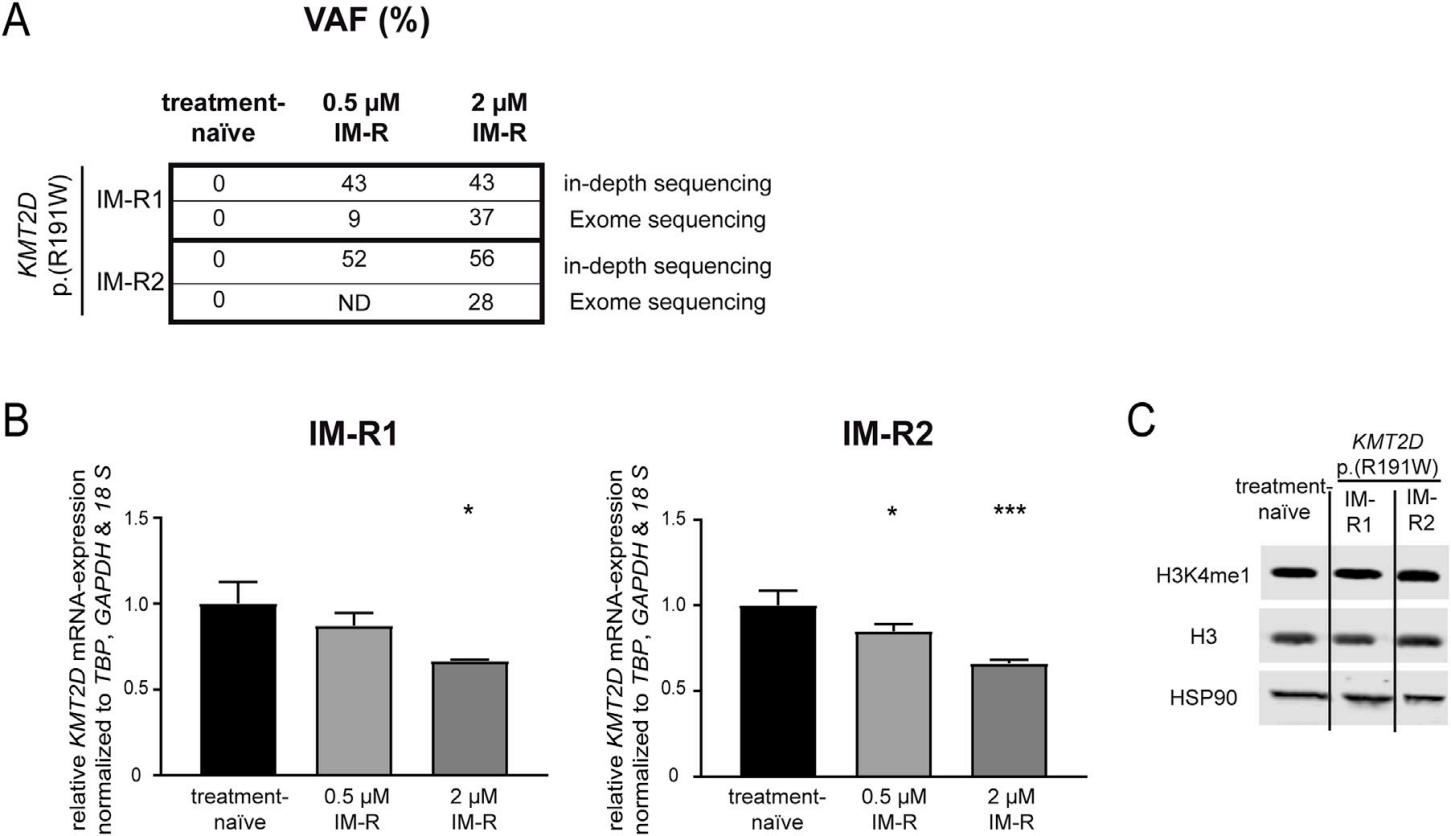
*KMT2D* p.(Arg191Trp) variant acquisition and expression in imatinib resistance. **(A)** Variant allele frequencies (VAF) of the *KMT2D* p. (Arg191Trp) variant in imatinib-resistant cell lines determined by exome or in-depth sequencing of K-562 cells resistant against 0.5 or 2 µM imatinib. **(B)** *KMT2D* mRNA expression in imatinib-resistant cells harboring *KMT2D* p. (Arg191Trp) measured by RT-qPCR and normalized to *TBP*, *GAPDH* and *18 S* and treatment-naïve cells. **(C)** Protein levels of H3K4me1, Histon 3 (H3) and HSP90 in treatment-naïve and imatinib-resistant cells harboring *KMT2D* p. (Arg191Trp). Statistical analyses were performed using One-way ANOVA followed by Dunnett’s tests. Error bars indicate standard deviation. N = 3. *: p < 0.05, ***: p < 0.001. IM, imatinib; ND, no data; R, resistant.

First, genetic variants contributing to imatinib resistance in an in vitro-K-562 CML cell line model were analyzed by exome sequencing. In two out of seven imatinib-resistant biological replicate cell lines that did not harbor BCR::ABL1 mutations, the *KMT2D* variant p. (Arg191Trp) (NM_003482) was recurrently detected with allele frequencies of 27% and 37%, respectively ((Kaehler et al., 2023), unpublished data, Figure 1A), while this variant was not detected in treatment-naïve K-562 cells. The presence of this variant was confirmed in these two cell lines by in-depth sequencing, as it was present in 43% and 52% of cells resistant to low dose imatinib (0.5 µM) and 43% and 56% cells resistant against high dose imatinib (2 μM, Figure 1A). This raised the question on the role of this gene and this particular *KMT2D* variant in imatinib resistance. In silico prediction revealed a CADD score of 29.6 and a PolyPhen score of 0.999 indicating a detrimental effect on KMT2D protein function. Thus, the *KMT2D* mRNA expression was analyzed and found to be significantly downregulated in both imatinib-resistant cell lines harboring the *KMT2D* p. (Arg191Trp) variant compared to treatment-naïve cells (R1: 2 µM IM: −33.3%, p = 0.03; R2: 0.5 µM IM: −15.1%, p = 0.03; 2 µM IM: −33.9%, p < 0.001, Figure 1B). In imatinib-resistant cell line replicates not carrying *KMT2D* variants, *KMT2D* mRNA was not differentially reduced, but even significantly upregulated in one replicate (Supplementary Figure S1). As KMT2D regulates methylation of H3K4, protein levels of histon 3 (H3) and its methylated form H4K4me1 were investigated, but did not reveal significant changes in the methylation between treatment-naïve and *KMT2D*-variant imatinib-resistant cells (Figure 1C).

### 3.2 Knockdown of *KMT2D* expression impairs the response to imatinib

**FIGURE 2:**
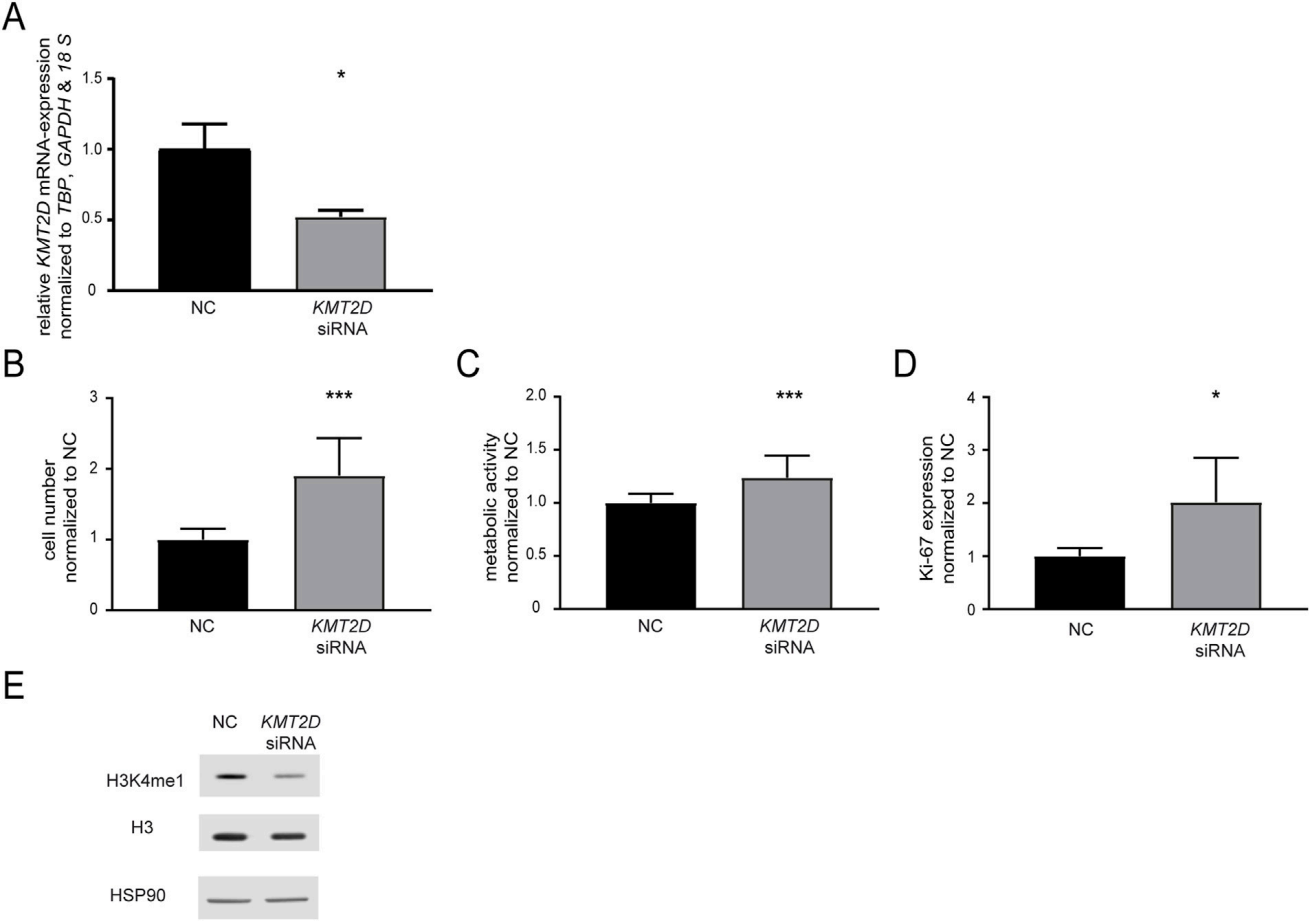
Knockdown of *KMT2D* hampers the response to imatinib treatment. **(A)** *KMT2D* mRNA expression after transfection with an *KMT2D*-targeting siRNA or siRNA negative control (NC) analyzed by RT-qPCR and normalized to *TBP*, *GAPDH*, *18 S* and NC. **(B–D)** Cell fitness after *KMT2D* knockdown under treatment with 2 µM imatinib analyzed on the level of **(B)** cell numbers **(C)** metabolic activity and **(D)** Ki-67 expression. **(E)** Protein levels of H3K4me1, histone H3 and HSP90 after knockdown of *KMT2D*. N = 3. Statistical analyses were performed using student’s tests. Error bars indicate standard deviation. N = 3. *: p < 0.05, ***: p < 0.001.

In a next step, the effect of KMT2D downregulation on imatinib susceptibility was analyzed by a siRNA-mediated knockdown. After successful knockdown of *KMT2D* (p = 0.02, Figure 2A), the cells were exposed to imatinib and cellular fitness was investigated. A significant increase in the cell number (90.6%, p < 0.001), metabolic activity (23.9%, p < 0.001) and proliferation rates (Ki-67-expression: 2.0-fold, p = 0.01) in the *KMT2D* knockdown was observed compared to negative control-transfected cells (Figures 2B–D). In addition, methylation of H3K4 was analyzed after silencing of KMT2D, revealing a slight decrease in H3K4me1 compared to Histone 3 and HSP90 levels (Figure 2E). This indicates that CML cells benefit from the loss of *KMT2D* expression under imatinib exposure, while histone methylation is reduced.

### 3.3 Rescue of *KMT2D* expression in imatinib-resistant CML cells restores imatinib susceptibility

**FIGURE 3:**
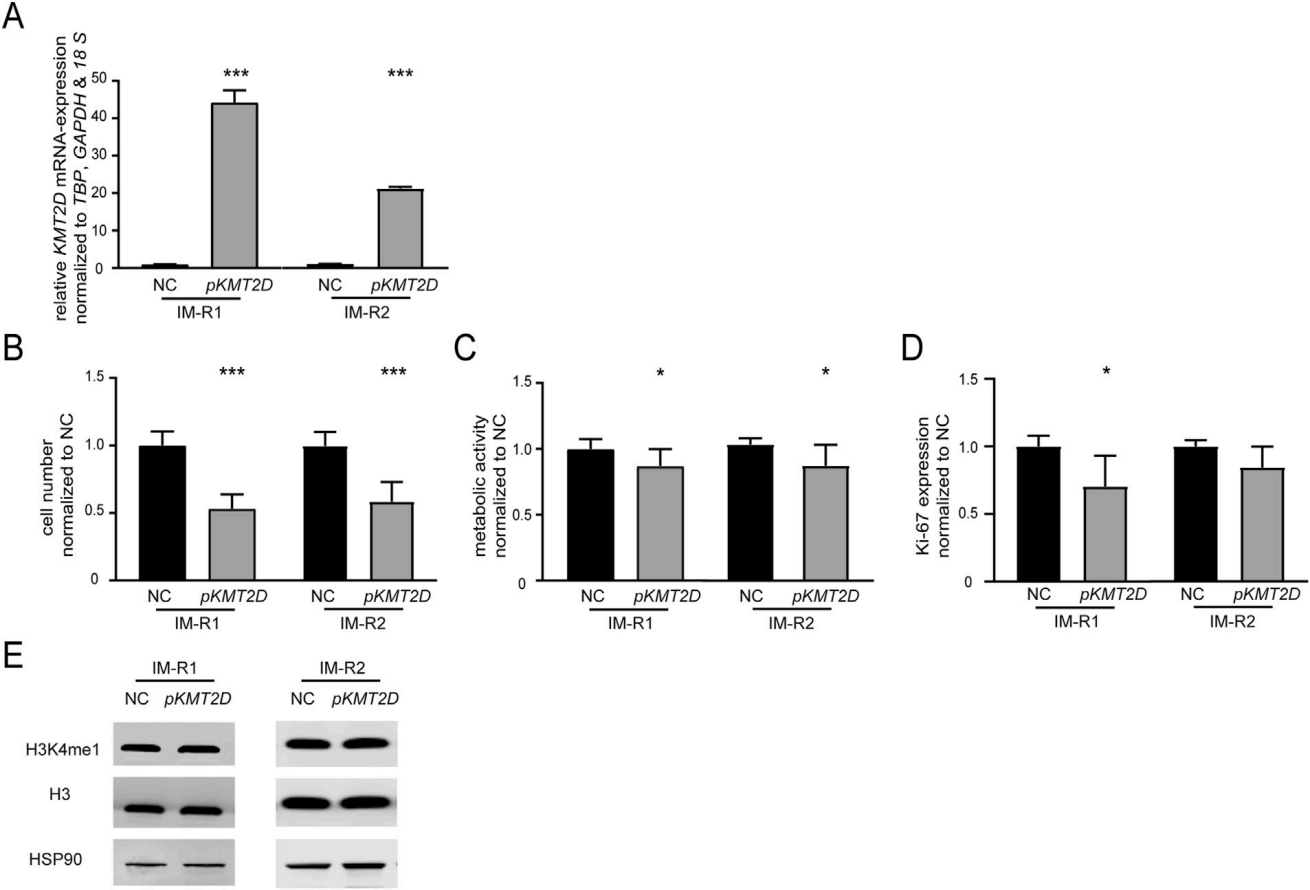
Restoration of *KMT2D* expression improves the response to imatinib in imatinib-resistant cells. **(A)** *KMT2D* mRNA expression after rescue of *KMT2D* in two imatinib-resistant cell lines harboring *KMT2D* p. (Arg191Trp) analyzed by RT-qPCR. Data was normalized to *TBP*, *GAPDH*, *18 S* and the respective empty vector negative control transfection (NC). **(B–D)** Cell fitness after rescue of *KMT2D* expression analyzed by **(B)** cell numbers, **(C)** metabolic activity and **(D)** Ki-67 expression after imatinib exposure. **(E)** Protein levels of H3K4me1 and histone H3 compared to HSP90 after restoration of *KMT2D* expression. Statistical analyses were performed using student’s tests. Error bars indicate standard deviation. N = 3. *: p < 0.05, ***: p < 0.001. NC: negative control.

As *KMT2D* expression was significantly downregulated in imatinib-resistant cell lines harboring the p. (Arg191Trp) variant, we were interested whether restoration of its expression by transfection of a *KMT2D*-encoding plasmid in these cell lines would increase imatinib-sensitivity (both: p < 0.001, Figure 3A). After *KMT2D* rescue, exposure to imatinib led to a reduction of cell numbers (IM-R1: −46.6%, p < 0.001, IM-R2: −41.2%, p < 0.001) and metabolic activities (IM-R1: −12.9%, p = 0.02, IM-R2: −15.7%, p = 0.02) compared to the empty vector control transfection. These findings indicate a restored susceptibility towards imatinib in both resistant cell lines (Figure 3B,C). However, proliferation rates were only significantly reduced in IM-R1 (−29.7%, p = 0.03, Figure 3D). In addition, analysis of H3K4 methylation did not reveal any changes (Figure 3E).

### 3.4 *KMT2D* p. (Arg191Trp) impairs the response to imatinib

**FIGURE 4:**
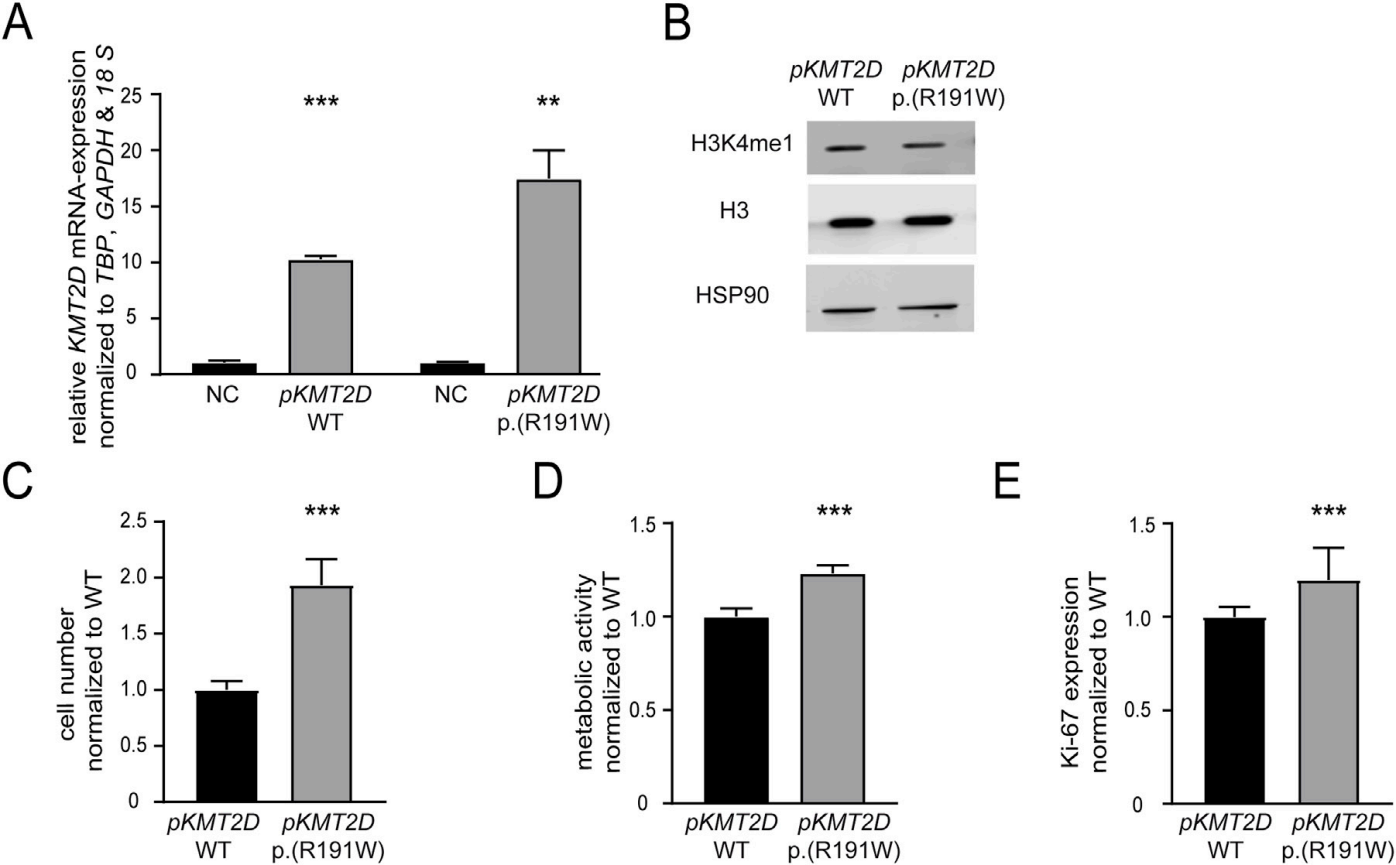
The presence of the *KMT2D* p.(Arg191Trp) variant promotes imatinib resistance. Transfection of treatment-naïve K-562 cells with *KMT2D* wild-type (WT) or p. (Arg191Trp). **(A)** RT-qPCR of *KMT2D* compared to *TBP*, *GAPDH*, *18 S* and normalized to the respective empty vector negative control (NC). **(B)** Protein levels of H3K4me1 and histone H3 compared to HSP90. **(C–E)** Cell fitness after transfection of *KMT2D* WT and p. (Arg191Trp) under imatinib treatment. **(C)** Cell numbers, **(D)** metabolic activity and **(D)** Ki-67 expression after exposure to 2 µM imatinib. Statistical analyses were performed using student’s tests. Error bars indicate standard deviation. N = 3. *: p < 0.05, **: p < 0.01, ***: p < 0.001. NC: negative control, *pKMT2D*: plasmid encoding *KMT2D* wild-type or p. (Arg191Trp).

Our previous findings suggested that the absence of *KMT2D* would be favorable for the development of imatinib resistance. In addition, it also pointed to a detrimental effect of the p. (Arg191Trp) variant on the KMT2D protein function. However, as the *KTM2D* variant’s effect on the protein function was still unclear, transfection experiments were performed to compare H3K4-methylation, cell numbers, proliferation and metabolic activity of either *KMT2D* wild-type or p. (Arg191Trp) in treatment-naïve K-562 cells (WT: p < 0.001; p. (R191W): p = 0.003, Figure 4A). In the presence of the KMT2D variant, methylation of H3K4 was slightly decreased compared to the KTM2D wild-type (Figure 4B). Under imatinib treatment, the presence of *KMT2D* p. (Arg191Trp) led to a significant increase in cell number compared to wild-type *KMT2D* (93%, p < 0.001, Figure 4C). In addition, metabolic activities (23%, p < 0.001) and proliferation rates (19.7%, p = 0.04), were also significantly increased under imatinib treatment (Figures 4D,E). These data confirm that the *KMT2D* p. (Arg191Trp) variant augments the development of imatinib resistance in CML.

**FIGURE 5:**
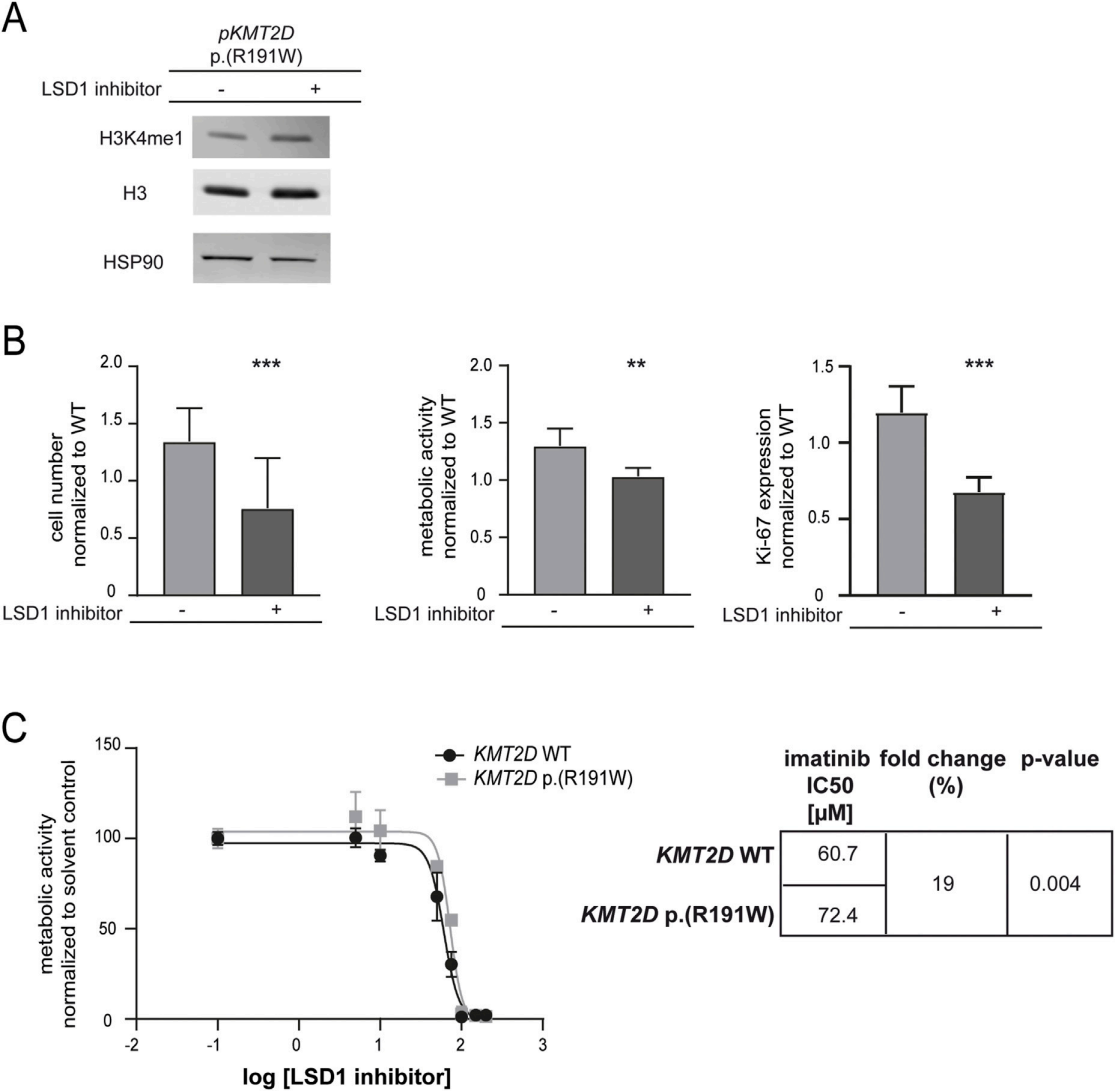
Sustained methylation of histone 3 compensates *KMT2D* p.(Arg191Trp)-mediated decrease in imatinib response. **(A)** Protein levels of H3K4me1 and histone H3 compared to HSP90. Depicted is one blot out of N = 3. **(B)** Cell fitness under imatinib treatment (2 µM) after transfection of *KMT2D* p. (Arg191Trp) in the presence of the HDMT inhibitor LSD1 inhibitor II (LSD1 inhibitor) on the level of cell numbers, metabolic activity and Ki-67 expression compared to solvent controls and normalized to *KMT2D* wild-type (WT). **(C)** IC50 values determined by metabolic activities of imatinib-resistant cells harboring *KMT2D* WT (black) or p. (Arg191Trp) (grey) analyzing two biological replicates, respectively. IC50 values were determined by non-linear regression with variable slope. Statistical analyses were performed using student’s tests. Error bars indicate standard deviation. N = 3. **: p < 0.01, ***: p < 0.001.

To restore the reduced methylation of H3K4 caused by the potential loss-of-function *KMT2D* p. (Arg191Trp) variant, the lysine-specific histone demethylase (LSD1) counteracting KMT2D function was inhibited. As expected, methylation of H3K4 was slightly increased under treatment with the LSD1 inhibitor in cells carrying the *KMT2D* variant (Figure 5A). Subsequently, the effect on imatinib susceptibility in these cells was analyzed. LSD1 inhibition led to a significant decrease in cell number (−26.7%, p < 0.001), metabolic activity (−58.4%, p = 0.007) and proliferation (−52.2%, p < 0.001, Figure 5B). Moreover, to assess whether LSD1 inhibition alters imatinib sensitivity in the context of the *KMT2D* variant, we compared the IC50 values of imatinib in the presence of the LSD1 inhibitor. Cells harboring the KMT2D variant exhibited a significantly higher IC50 compared to wild-type (imatinib IC50: 60.7 µM vs. 72.4 µM, 19%, p = 0.004, Figure 5C) indicating a reduced imatinib susceptibility in the presence of the *KMT2D* variant.

### 3.5 Epigenetic modulators in imatinib resistance and the response to imatinib

**FIGURE 6:**
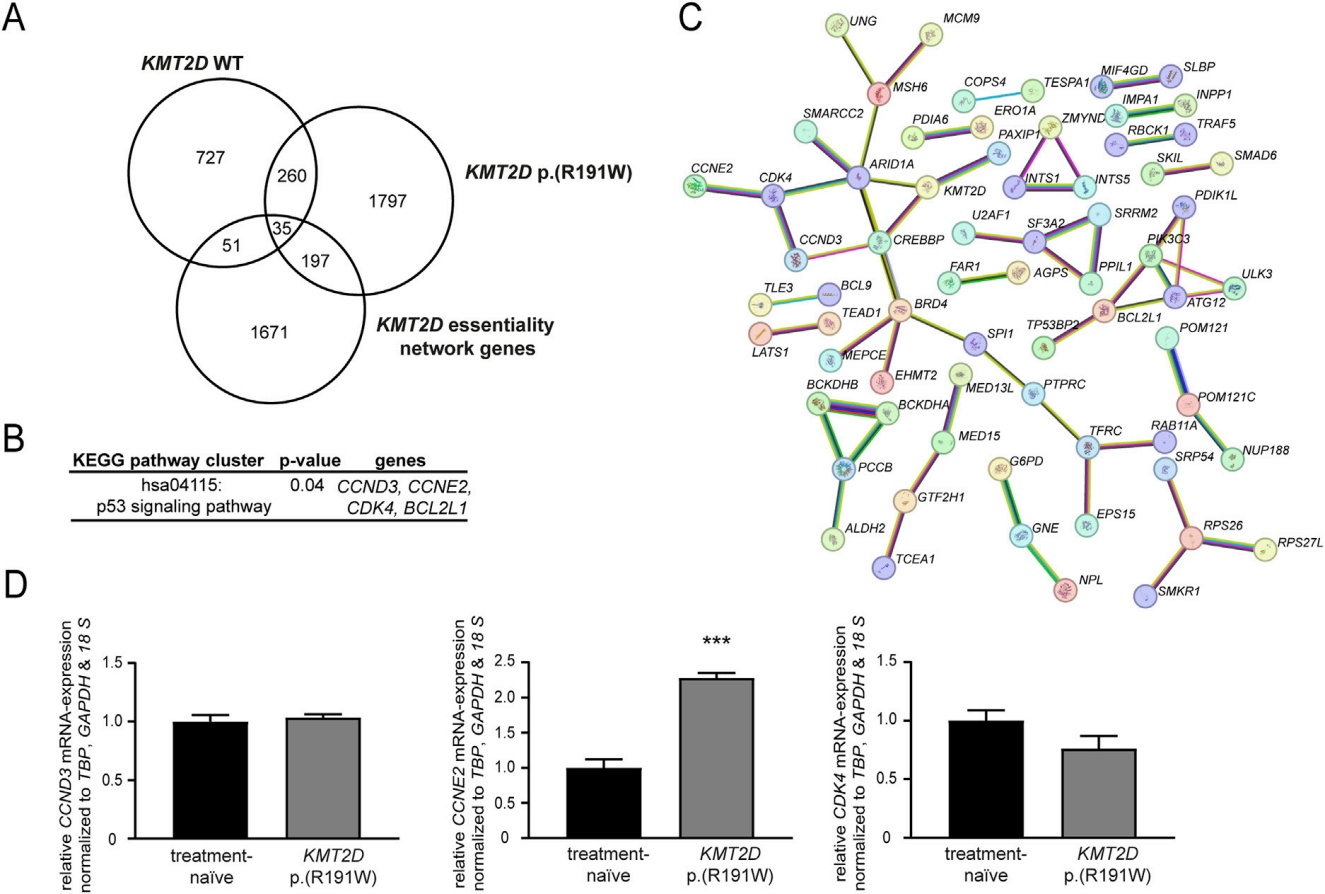
Identification of putative *KMT2D* target genes. **(A–C)** Genome-wide expression data from imatinib-resistant K-562 cells harboring *KMT2D* wild-type (WT) or p. (Arg191Trp) were compared to the *KMT2D* essentiality network (1954 genes) obtained from Takemon et al. (2024). **(A)** Overlap of differentially expressed genes in imatinib-resistant cells harboring *KMT2D* WT or p. (Arg191Trp). **(B)** KEGG pathway cluster and **(C)** STRING analysis of the 197 genes differentially expressed in *KMT2D* p. (Arg191Trp) belonging to the *KMT2D* essentiality network (including *KMT2D*, high confidence settings). Pink: experimentally validated, turquoise: from curated database, green: gene neighborhood, red: gene fusions, blue: gene co-occurrence, yellow: text mining, black: co-expression, light blue: protein homology. **(D)** mRNA expression of *CCND3*, *CCNE2* and *CDK4* in imatinib-resistant cells harboring *KMT2D* p. (Arg191Trp) compared to treatment-naïve cells analyzed by RT-qPCR normalized to the housekeeping genes *TBP*, *GAPDH*, and *18 S.* N = 3. ***: p < 0.001.

To get a deeper insight into mechanisms underlying the loss of imatinib-susceptibility in presence of *KMT2D* p. (Arg191Trp), genome-wide expression data from imatinib-resistant cells harboring *KMT2D* wild-type or p. (Arg191Trp) were obtained and compared to imatinib-sensitive K-562 cells ((Kaehler et al., 2022; Kaehler et al., 2023); GSE227347, GSE203342). These expression profiles were compared with the *KMT2D* essentiality network, a list of 1954 genes from Takemon et al. (Takemon et al., 2024), to identify potential genes targeted by *KMT2D*. In *KMT2D* wild-type imatinib-resistant cells, 51 genes were detected, while in *KMT2D* variant cells 197 genes were found (Figure 6A). By subsequent KEGG pathway cluster analysis, an enrichment with genes involved in the p53 signaling pathway was detected (Figure 6B), also showing an interaction of the respective genes in the STRING annotation (Figure 6C). These upregulated genes were the cyclins *CCND3* (3.2-fold) and *CCNE2* (4.3-fold) and the cyclin-dependent kinase *CDK4* (2.2-fold enriched in the imatinib-resistant *KMT2D* variant cells). As these genes are putative indirect interaction partners of KMT2D via the chromatin-remodeling complex protein ARID1A or the co-activator of transcription CREBBP, the question arose if their expression is influenced by the *KMT2D* variant. In subsequent analysis of mRNA expression levels, upregulation of *CCNE2* (2.2-fold, p < 0.001) in imatinib-resistant cells harboring the *KMT2D* variant compared to treatment-naïve cells was confirmed, while expression of *CCND3* and *CDK4* was not altered (Figure 6D). These findings indicate that proliferation of cells harboring the *KMT2D* variant could be mediated by upregulation of *CCNE2*.

**FIGURE 7:**
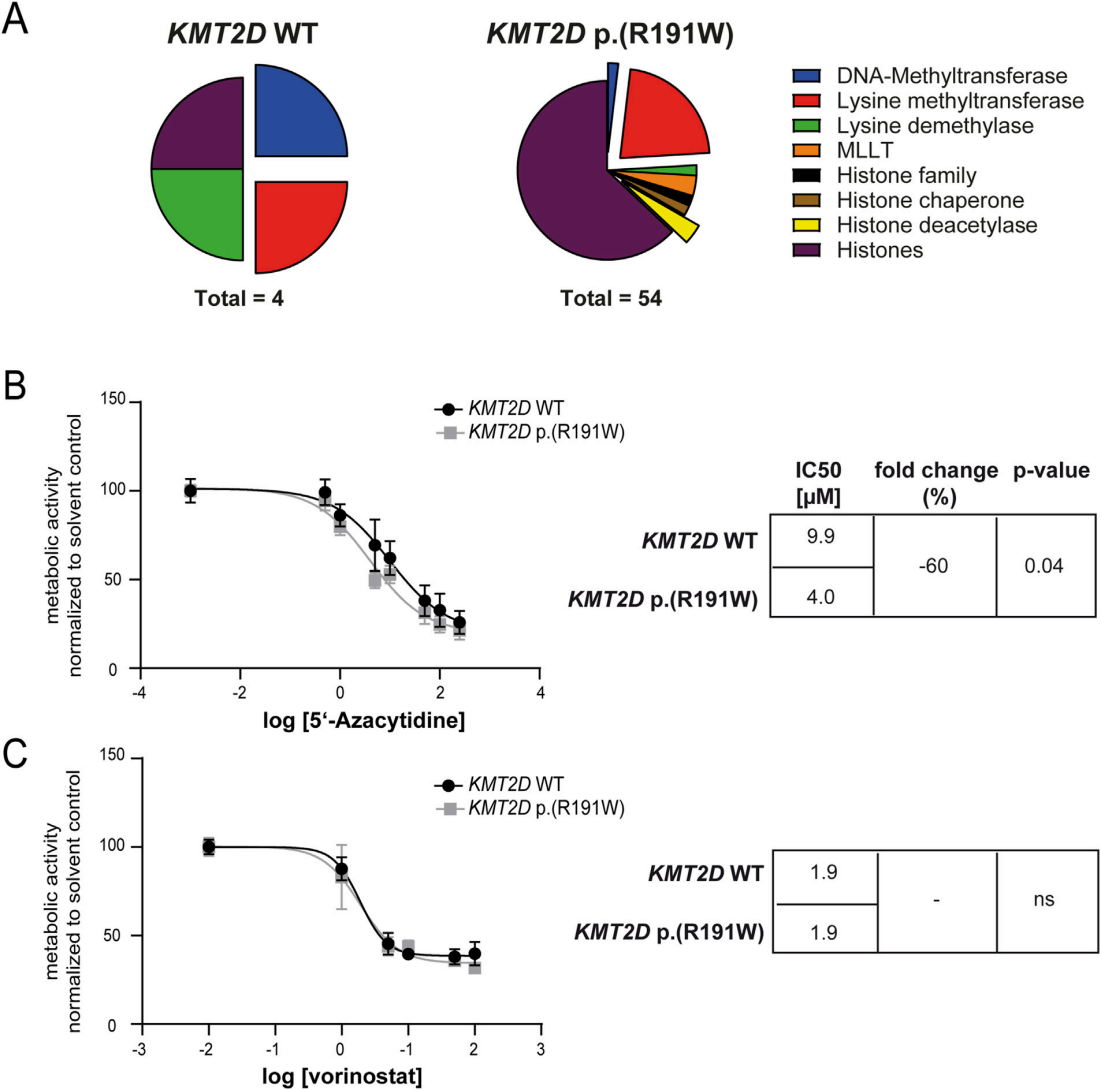
Epigenetic modifiers and their inhibition in imatinib-resistant cells harboring *KMT2D* wild-type or p.(Arg191Trp). **(A)** Pie charts of differentially expressed epigenetic modifiers and histones in imatinib-resistant K-562 cells harboring *KMT2D* wild-type (WT) or p. (Arg191Trp). Blue: DNA-Methyltransferases, Red: Lysine methyltransferases, Green: Lysine demethylases, Orange: MLLT, Black: Histone family, Brown: Histone chaperone, Yellow: Histone deacetylase, purple: Histones. **(B–C)** Metabolic activity of imatinib-resistant cell lines harboring *KMT2D* WT or p. (Arg191Trp) in the presence of **(B)** 5′-azacytidine or **(C)** vorinostat with the respective IC50 values. Data were normalized to the respective solvent control. IC50 values were determined by non-linear fit. Error bars indicate standard deviation. N = 3.

Besides KMT2D, deregulation of other epigenetic factors in imatinib-resistant CML was analyzed. Thus, genome-wide gene expression from imatinib-resistant and treatment-naïve K-562 biological replicate cell lines harboring *KMT2D* wild-type or p. (Arg191Trp) derived from the GSE203342 and GSE227347 datasets were compared and filtered for significant deregulation of genes encoding epigenetic modulators and histones. The number of differentially expressed genes varied between four and 54 between *KMT2D* wild-type and variant (Figure 7A). As *DNMT* or *HDAC* genes were differentially deregulated between *KMT2D* wild-type or variant imatinib-resistant cells, this raised the question on the efficiency of epigenetic modulation in the presence and absence of the *KMT2D* variant. Thus, the cell lines were exposed to the DNMT inhibitor 5′-azacytidine or the HDAC inhibitor vorinostat. Under treatment with 5′-azacytidine, the imatinib IC50 was significantly reduced in *KMT2D* p. (Arg191Trp) cells compared to wild-type cells (−60%, p = 0.04, Figure 7B), while the HDAC inhibitor vorinostat did not alter the response to imatinib (Figure 7C).

## 4 Discussion

In the present study, the role of *KMT2D* and its variant p. (Arg191Trp) were analyzed in imatinib-resistant CML cell lines *in vitro*. We found that the *KMT2D* variant was recurrently acquired in imatinib resistance, while its expression was reduced in the respective cell lines. Applying transfection experiments, it could be confirmed that the presence of the variant promotes imatinib resistance, which could be mimicked by siRNA-mediated knockdown of *KMT2D* expression. In addition, in resistant cells the imatinib susceptibility could be restored by rescue of *KMT2D* expression.

For our study, we used concentrations of 0.5 and 2 µM imatinib to study the effects of *KMT2D* and its variant on imatinib susceptibility and resistance. These concentrations reflect the range of 0.3 and 3.4 µM determined in plasma of CML patients undergoing imatinib therapy. The ideal plasma concertation is 1 μg/mL (1.7 µM) (De Kogel and Schellens, 2007; Picard et al., 2007). This dose range also reflects the facts that variable biotransformation or comorbidities lead to interindividual differences in plasma concentrations and that distribution into deeper body compartments, such as the bone marrow is lower (Leveque and Maloisel, 2005; Peng et al., 2005; Cortes et al., 2009).

Besides *KMT2C*, *KMT2D* belongs to one of the most frequently mutated genes in cancer and is considered as a tumor suppressor gene displaying negative effects on cell growth (Kandoth et al., 2013; Lawrence et al., 2014). A study from Liu et al. revealed somatic *KMT2D* mutations in about 19% of patients suffering from diffuse large B cell lymphoma (DLBCL) promoting tumor progression (Liu Q. X. et al., 2024). In addition, in 16% of cases with childhood medulloblastoma, *KMT2D* deficiency was found with the majority being protein alternating missense mutations or truncations (Parsons et al., 2011). Also, in head and neck squamous cell carcinoma (HNSCC), *KMT2D* was identified as a tumor suppressor gene promoting cell growth through increasing glycolysis (Liu W. et al., 2024). In general, heterozygote loss of *KMT2D* has not been considered as the initial disease-causing driving force of cancer, as studies have not revealed spontaneous tumor formation after deletion of the gene (Rao and Dou, 2015). However, loss of *KMT2D* might be linked to tumor progression or development of therapy resistance, as observed here.

The KMT2D protein contains of *N*-terminal two plant homology domain (PHD) cluster and a *C*-terminal SET domain (Froimchuk et al., 2017). According to Rao et al., pathogenic *KMT2D* mutations mainly affect the SET domain (37.0%) and the PHD domains (60%) and in most cases result in protein altering truncations (Rao and Dou, 2015). Regarding the *KMT2D* variant c.571C>T, p. (Arg191Trp) recurrently detected here, this particular variant (s1555198522) is considered to be associated with hereditary Kabuki syndrome (Bogershausen and Wollnik, 2013; Boniel et al., 2024). However, knowledge about this variant in oncogenic diseases is still missing. According to UniProt, the p. Arg191 residue is located in the histone-binding PHD-type 1 zinc-finger motif, which ranges from the amino acids 170 to 218 (UniProt, 2025). Thus, amino acids exchanges in this region could likely affect the function of the PHD domain and thereby lead to loss-of-function, also indicated by the respective variant prediction scores. However, for the same residue, the c.572G>A, p. (Arg191Gln) variant, which results in an amino acid exchange to glutamine (rs548930191), the effect on the KMT2D protein seem to be benign (Kopanos et al., 2019). In the present study, we demonstrate that *KMT2D* p. (Arg191Trp) was acquired in an imatinib-resistant CML cell line and its introduction into treatment-naïve K-562 cells displayed similar effects as siRNA-mediated *KMT2D* downregulation impairing the response to imatinib. In addition, mono-methylation of H3K4 was reduced in both cases, while sustained methylation by inhibition of H3K4-methylation restored the response to imatinib in the presence of KMT2D p. (Arg191Trp). These findings indicate *KMT2D* p. (Arg191Trp) as a loss-of-function variant affecting KMT2D protein function. In CML, *KMT2D* (but also *KMT2C*) downregulation was found to correlate with the CML phases, and thus, disease progression, but also with the response of CML patients (Rabello et al., 2018). These findings stand in line with our present in vitro-study, where the response to imatinib could be improved by rescue of *KMT2D* expression in imatinib-resistant cells.

To analyze the genes affected by the *KMT2D* p. (Arg191Trp) variant, genome-wide expression changes in imatinib-resistant cells harboring the variant were analyzed and compared to the KMT2D essentiality network (Takemon et al., 2024). Of the 197 genes detected from this network, four were enriched in the p53-signaling pathway. However, only upregulation of cyclin E2 (*CCNE2*) expression could be confirmed by RT-qPCR. As a cell cycle progressor, CCNE2 has been shown to promote proliferation of cancer cells, as demonstrated, e.g. in prostate or ovarian cancer (Xie et al., 2017; Liu et al., 2020; Fagundes and Teixeira, 2021). The upregulation of *CCNE2* in cells harboring *KMT2D* p. (Arg191Trp) with loss of KMT2D function potentially promotes the development of resistance against imatinib in CML. However, to provide more insights into this, the KMT2D network of treatment-naïve CML cells expressing *KMT2D* wildtype or p. (Arg191Trp) needs to be analyzed to exclude potential effects due to adaptions of gene expression in TKI resistance. Overall, our data as well as in silico-analyses indicate that the *KMT2D* p. (Arg191Trp) variant results in a loss-of-function of the protein. As a potential result, the histone methylation of target genes, among them genes of the p53 signaling pathway, is decreased. This leads to their upregulation, as demonstrated, e.g. for *CCNE2*, and subsequently, CML progression and the development of TKI resistance.

Thus, the question arises if epigenetic modulators would be beneficial to overcome TKI resistance in CML. In our study, inhibition of DNMTs by 5′-azacytidine led to a slight increase in imatinib susceptibility in cells harboring *KMT2D* p. (Arg191Trp), while inhibition of HDACs by vorinostat did not display any effects (regardless from the presence of *KMT2D* mutations or deregulation of other epigenetic modifiers). This indicates DNMT inhibition as potential strategy to overcome TKI resistance. In a study on CML evolution, it has been demonstrated that epigenetic reprogramming and aberrant DNA methylation contributes to CML progression (Amabile et al., 2015; Bugler et al., 2019). Thus, it was shown that the concomitant use of decitabine and imatinib may result in improved TKI responses (San Jose-Eneriz et al., 2009). It has been demonstrated that low-dose decitabine can be effective in CML taking advantage of its demethylating properties, while reducing cytotoxicity (Yang et al., 2006). In addition, other TKI combinations with BCL-2- inhibitors, e.g. venetoclax, or HDAC inhibitors, e.g. panobinostat, were experimentally tested and showed promising results (Amir and Javed, 2021).

A limitation of the present study is the fact that these findings are based on an in vitro-model of imatinib-resistant CML cells. While the *KMT2D* p. (Arg191Trp) variant has been recurrently detected in biological replicates of imatinib resistance, their occurrence needs to be further evaluated in a clinical study in CML patients. Regarding the efficacy of downstream target inhibition, e.g. CCNE2 inhibitors, to overcome imatinib resistance, further studies are necessary to address the role of *KMT2D* in therapy resistant CML. This is also the case for the potential use of epigenetic modifiers in combinatory treatment regimens in CML.

## 5 Conclusion

Overall, our data demonstrate that *KMT2D* and its variant p. (Arg191Trp), which seems to result in a protein altering loss-of-function variant, are involved in the development of TKI resistance in CML in an in vitro-model. The variant itself does not seem to be the single driver mutation in cancer, but as *KMT2D* variants are recurrently acquired in cancer, the loss of *KMT2D* pronounced tumor progression, or as observed here, therapy resistance potentially due to increased genetic instability and epigenetic reprogramming. These findings indicate *KMT2D* as a potential target vulnerability for combinational therapy in CML, but also in other cancer entities. Further, *KMT2D* status could be a potential biomarker for the treatment of CML with TKIs.

## Data Availability

The original contributions presented in the study are included in the article/Supplementary Material, further inquiries can be directed to the corresponding author.

## References

[B1] Amabile G. Di Ruscio A. Muller F. Welner R. S. Yang H. Ebralidze A. K. (2015). Dissecting the role of aberrant DNA methylation in human leukaemia. Nat. Commun. 6, 7091. 10.1038/ncomms8091 25997600 PMC4443494

[B2] Amir M. Javed S. (2021). A review on the therapeutic role of TKIs in case of CML in combination with epigenetic drugs. Front. Genet. 12, 742802. 10.3389/fgene.2021.742802 34745216 PMC8569791

[B3] Bixby D. Talpaz M. (2011). Seeking the causes and solutions to imatinib-resistance in chronic myeloid leukemia. Leukemia 25, 7–22. 10.1038/leu.2010.238 21102425

[B4] Bogershausen N. Wollnik B. (2013). Unmasking Kabuki syndrome. Clin. Genet. 83, 201–211. 10.1111/cge.12051 23131014

[B5] Boniel S. Krajewska M. Pyrzak B. Paluchowska M. Majcher A. Zarlenga M. (2024). Clinical and molecular characteristics of Kabuki syndrome patients with missense variants-novel features and literature review. Front. Genet. 15, 1402531. 10.3389/fgene.2024.1402531 39104744 PMC11298422

[B6] Bruhn O. Lindsay M. Wiebel F. Kaehler M. Nagel I. Bohm R. (2020). Alternative polyadenylation of ABC transporters of the C-family (ABCC1, ABCC2, ABCC3) and implications on posttranscriptional micro-RNA regulation. Mol. Pharmacol. 97, 112–122. 10.1124/mol.119.116590 31757862

[B7] Bugler J. Kinstrie R. Scott M. T. Vetrie D. (2019). Epigenetic reprogramming and emerging epigenetic therapies in CML. Front. Cell Dev. Biol. 7, 136. 10.3389/fcell.2019.00136 31380371 PMC6652210

[B8] Cortes J. E. Egorin M. J. Guilhot F. Molimard M. Mahon F. X. (2009). Pharmacokinetic/pharmacodynamic correlation and blood-level testing in imatinib therapy for chronic myeloid leukemia. Leukemia 23, 1537–1544. 10.1038/leu.2009.88 19404318

[B9] De Kogel C. E. Schellens J. H. (2007). Imatinib. Oncologist 12, 1390–1394. 10.1634/theoncologist.12-12-1390 18165615

[B10] Druker B. J. Tamura S. Buchdunger E. Ohno S. Segal G. M. Fanning S. (1996). Effects of a selective inhibitor of the Abl tyrosine kinase on the growth of Bcr-Abl positive cells. Nat. Med. 2, 561–566. 10.1038/nm0596-561 8616716

[B11] Fagundes R. Teixeira L. K. (2021). Cyclin E/CDK2: DNA replication, replication stress and genomic instability. Front. Cell Dev. Biol. 9, 774845. 10.3389/fcell.2021.774845 34901021 PMC8652076

[B12] Ford D. J. Dingwall A. K. (2015). The cancer COMPASS: navigating the functions of MLL complexes in cancer. Cancer Genet. 208, 178–191. 10.1016/j.cancergen.2015.01.005 25794446

[B13] Froimchuk E. Jang Y. Ge K. (2017). Histone H3 lysine 4 methyltransferase KMT2D. Gene 627, 337–342. 10.1016/j.gene.2017.06.056 28669924 PMC5546304

[B14] Hochhaus A. Larson R. A. Guilhot F. Radich J. P. Branford S. Hughes T. P. (2017). Long-term outcomes of imatinib treatment for chronic myeloid leukemia. N. Engl. J. Med. 376, 917–927. 10.1056/nejmoa1609324 28273028 PMC5901965

[B15] Huang Da W. Sherman B. T. Lempicki R. A. (2009). Systematic and integrative analysis of large gene lists using DAVID bioinformatics resources. Nat. Protoc. 4, 44–57. 10.1038/nprot.2008.211 19131956

[B16] Kaehler M. Cascorbi I. (2023). Molecular mechanisms of tyrosine kinase inhibitor resistance in chronic myeloid leukemia. Handb. Exp. Pharmacol. 280, 65–83. 10.1007/164_2023_639 36882601

[B17] Kaehler M. Ruemenapp J. Gonnermann D. Nagel I. Bruhn O. Haenisch S. (2017). MicroRNA-212/ABCG2-axis contributes to development of imatinib-resistance in leukemic cells. Oncotarget 8, 92018–92031. 10.18632/oncotarget.21272 29190894 PMC5696160

[B18] Kaehler M. Dworschak M. Rodin J. P. Ruemenapp J. Vater I. Penas E. M. M. (2021). ZFP36L1 plays an ambiguous role in the regulation of cell expansion and negatively regulates CDKN1A in chronic myeloid leukemia cells. Exp. Hematol. 99, 54–64.e7. 10.1016/j.exphem.2021.05.006 34090970

[B19] Kaehler M. Litterst M. Kolarova J. Bohm R. Bruckmueller H. Ammerpohl O. (2022). Genome-wide expression and methylation analyses reveal aberrant cell adhesion signaling in tyrosine kinase inhibitor-resistant CML cells. Oncol. Rep. 48, 144. 10.3892/or.2022.8355 35730629 PMC9245083

[B20] Kaehler M. Osteresch P. Kunstner A. Vieth S. J. Esser D. Moller M. (2023). Clonal evolution in tyrosine kinase inhibitor-resistance: lessons from *in vitro*-models. Front. Oncol. 13, 1200897. 10.3389/fonc.2023.1200897 37384296 PMC10294234

[B21] Kandoth C. Mclellan M. D. Vandin F. Ye K. Niu B. Lu C. (2013). Mutational landscape and significance across 12 major cancer types. Nature 502, 333–339. 10.1038/nature12634 24132290 PMC3927368

[B22] Kopanos C. Tsiolkas V. Kouris A. Chapple C. E. Albarca Aguilera M. Meyer R. (2019). VarSome: the human genomic variant search engine. Bioinformatics 35, 1978–1980. 10.1093/bioinformatics/bty897 30376034 PMC6546127

[B23] Lawrence M. S. Stojanov P. Mermel C. H. Robinson J. T. Garraway L. A. Golub T. R. (2014). Discovery and saturation analysis of cancer genes across 21 tumour types. Nature 505, 495–501. 10.1038/nature12912 24390350 PMC4048962

[B24] Leveque D. Maloisel F. (2005). Clinical pharmacokinetics of imatinib mesylate. vivo (Athens, Greece) 19, 77–84. 15796158

[B25] Liu B. Qian D. Zhou W. Jiang H. Xiang Z. Wu D. (2020). A novel androgen-induced lncRNA FAM83H-AS1 promotes prostate cancer progression via the miR-15a/CCNE2 Axis. Front. Oncol. 10, 620306. 10.3389/fonc.2020.620306 33614501 PMC7890020

[B26] Liu Q. X. Zhu Y. Yi H. M. Shen Y. G. Wang L. Cheng S. (2024). *KMT2D* mutations promoted tumor progression in diffuse large B-cell lymphoma through altering tumor-induced regulatory T cell trafficking via FBXW7-NOTCH-MYC/TGF-β1 axis. Int. J. Biol. Sci. 20, 3972–3985. 10.7150/ijbs.93349 39113693 PMC11302885

[B27] Liu W. Cao H. Wang J. Elmusrati A. Han B. Chen W. (2024). Histone-methyltransferase KMT2D deficiency impairs the Fanconi anemia/BRCA pathway upon glycolytic inhibition in squamous cell carcinoma. Nat. Commun. 15, 6755. 10.1038/s41467-024-50861-5 39117659 PMC11310337

[B28] Livak K. J. Schmittgen T. D. (2001). Analysis of relative gene expression data using real-time quantitative PCR and the 2(-Delta Delta C(T)) Method. Methods (San Diego, Calif.) 25, 402–408. 10.1006/meth.2001.1262 11846609

[B29] Loh S. W. Ng W. L. Yeo K. S. Lim Y. Y. Ea C. K. (2014). Inhibition of euchromatic histone methyltransferase 1 and 2 sensitizes chronic myeloid leukemia cells to interferon treatment. PLoS One 9, e103915. 10.1371/journal.pone.0103915 25079219 PMC4117596

[B30] Lozzio C. B. Lozzio B. B. (1975). Human chronic myelogenous leukemia cell-line with positive Philadelphia chromosome. Blood 45, 321–334. 10.1182/blood.v45.3.321.321 163658

[B31] Milojkovic D. Apperley J. (2009). Mechanisms of resistance to imatinib and second-generation tyrosine inhibitors in chronic myeloid leukemia. Clin. Cancer Res. 15, 7519–7527. 10.1158/1078-0432.CCR-09-1068 20008852

[B32] Minciacchi V. R. Kumar R. Krause D. S. (2021). Chronic myeloid leukemia: a model disease of the past, present and future. Cells 10, 117. 10.3390/cells10010117 33435150 PMC7827482

[B33] Nowell P. C. Hungerford D. A. (1960). Chromosome studies on normal and leukemic human leukocytes. J. Natl. Cancer Inst. 25, 85–109. 14427847

[B34] Oliveros J. C. (2007). An interactive tool for comparing lists with Venn's diagrams.

[B35] Parsons D. W. Li M. Zhang X. Jones S. Leary R. J. Lin J. C. (2011). The genetic landscape of the childhood cancer medulloblastoma. Science 331, 435–439. 10.1126/science.1198056 21163964 PMC3110744

[B36] Peng B. Lloyd P. Schran H. (2005). Clinical pharmacokinetics of imatinib. Clin. Pharmacokinet. 44, 879–894. 10.2165/00003088-200544090-00001 16122278

[B37] Picard S. Titier K. Etienne G. Teilhet E. Ducint D. Bernard M. A. (2007). Trough imatinib plasma levels are associated with both cytogenetic and molecular responses to standard-dose imatinib in chronic myeloid leukemia. Blood 109, 3496–3499. 10.1182/blood-2006-07-036012 17192396

[B38] Rabello D. D. A. Ferreira V. Berzoti-Coelho M. G. Burin S. M. Magro C. L. Cacemiro M. D. C. (2018). MLL2/KMT2D and MLL3/KMT2C expression correlates with disease progression and response to imatinib mesylate in chronic myeloid leukemia. Cancer Cell Int. 18, 26. 10.1186/s12935-018-0523-1 29483845 PMC5819641

[B39] Rao R. C. Dou Y. (2015). Hijacked in cancer: the KMT2 (MLL) family of methyltransferases. Nat. Rev. Cancer 15, 334–346. 10.1038/nrc3929 25998713 PMC4493861

[B40] Rowley J. D. (1973). Letter: a new consistent chromosomal abnormality in chronic myelogenous leukaemia identified by quinacrine fluorescence and Giemsa staining. Nature 243, 290–293. 10.1038/243290a0 4126434

[B41] San Jose-Eneriz E. Agirre X. Jimenez-Velasco A. Cordeu L. Martin V. Arqueros V. (2009). Epigenetic down-regulation of BIM expression is associated with reduced optimal responses to imatinib treatment in chronic myeloid leukaemia. Eur. J. Cancer 45, 1877–1889. 10.1016/j.ejca.2009.04.005 19403302

[B42] Sherman B. T. Hao M. Qiu J. Jiao X. Baseler M. W. Lane H. C. (2022). DAVID: a web server for functional enrichment analysis and functional annotation of gene lists (2021 update). Nucleic Acids Res. 50, W216–W221. 10.1093/nar/gkac194 35325185 PMC9252805

[B43] Takemon Y. Pleasance E. D. Gagliardi A. Hughes C. S. Csizmok V. Wee K. (2024). Mapping *in silico* genetic networks of the KMT2D tumour suppressor gene to uncover novel functional associations and cancer cell vulnerabilities. Genome Med. 16, 136. 10.1186/s13073-024-01401-9 39578878 PMC11583415

[B44] Turrini E. Haenisch S. Laechelt S. Diewock T. Bruhn O. Cascorbi I. (2012). MicroRNA profiling in K-562 cells under imatinib treatment: influence of miR-212 and miR-328 on ABCG2 expression. Pharmacogenet Genomics 22, 198–205. 10.1097/FPC.0b013e328350012b 22241070

[B45] Uniprot C. Martin M. J. Orchard S. Magrane M. Adesina A. Ahmad S. (2025). UniProt: the universal protein knowledgebase in 2025. Nucleic Acids Res. 53, D609–D617. 10.1093/nar/gkae1010 39552041 PMC11701636

[B46] Waetzig V. Haeusgen W. Andres C. Frehse S. Reinecke K. Bruckmueller H. (2019). Retinoic acid-induced survival effects in SH-SY5Y neuroblastoma cells. J. Cell Biochem. 120, 5974–5986. 10.1002/jcb.27885 30320919

[B47] Xie L. Li T. Yang L. H. (2017). E2F2 induces MCM4, CCNE2 and WHSC1 upregulation in ovarian cancer and predicts poor overall survival. Eur. Rev. Med. Pharmacol. Sci. 21, 2150–2156. 28537669

[B48] Yang A. S. Doshi K. D. Choi S. W. Mason J. B. Mannari R. K. Gharybian V. (2006). DNA methylation changes after 5-aza-2'-deoxycytidine therapy in patients with leukemia. Cancer Res. 66, 5495–5503. 10.1158/0008-5472.CAN-05-2385 16707479

