# Supplementary material for: The role of the lysine histone methylase KMT2D in chronic myeloid leukemia
Source: Front Pharmacol. 2025 Sep 16;16:1652373. doi: 10.3389/fphar.2025.1652373 (PMC12481891; doi:10.3389/fphar.2025.1652373)

**Original Western Blot data**

**Fig. 1: *KMT2D* expression and presence of p.Arg191Trp in imatinib-resistant CML.** N = 3.

| Marker | treatment-naive | treatment-naive | treatment-  naive | IM-R1 | IM-R1 | IM-R1 | IM-R2 | IM-R2 | IM-R2 | unrelated | unrelated | unrelated |
| --- | --- | --- | --- | --- | --- | --- | --- | --- | --- | --- | --- | --- |

| 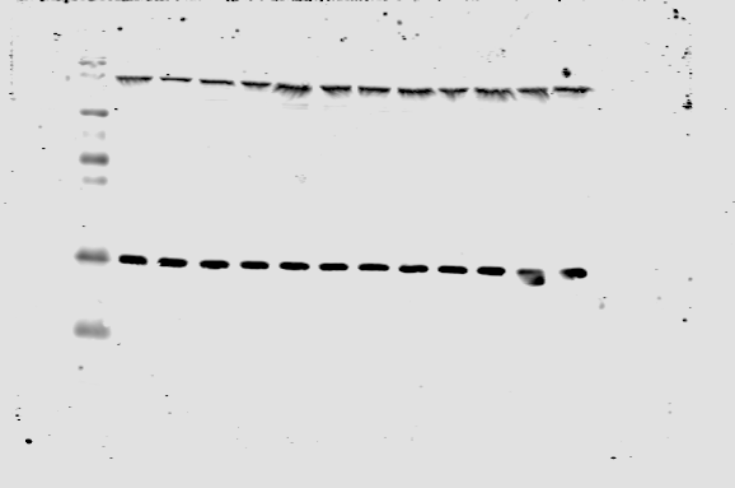 | **HSP90** |
| --- | --- |
|  | **H3K4** |
| 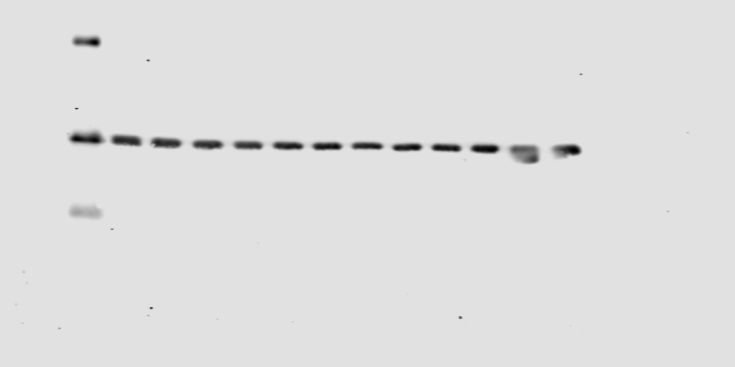 | **H3** |

**Fig. 2: Knockdown of *KMT2D* expression impairs the response to imatinib.** N = 4.

| Marker | NC | siRNA | NC | siRNA | NC | siRNA | NC | siRNA | Marker |
| --- | --- | --- | --- | --- | --- | --- | --- | --- | --- |

NC: negative control, siRNA: siRNA targeting *KMT2D*.

| 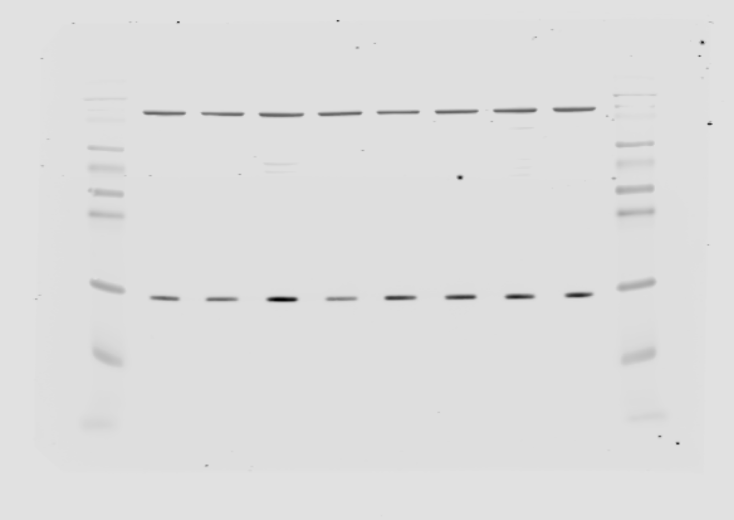 | **HSP90** |
| --- | --- |
|  | **H3K4** |
| 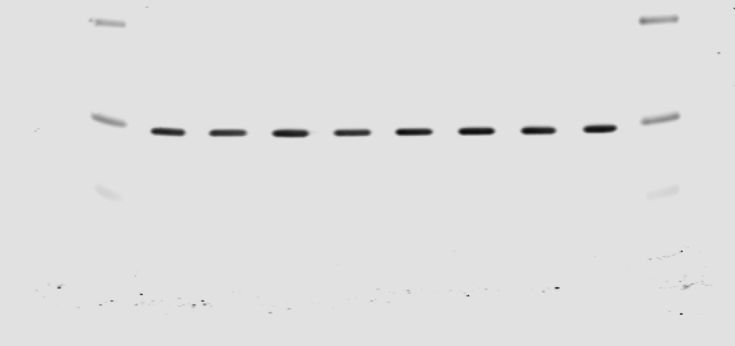 | **H3** |

**Fig. 3: Rescue of *KMT2D* expression in imatinib-resistant CML cells restores imatinib susceptibility.** N = 3 for two biological replicate cell lines (IM-R1, IM-R2).

**IM-R1**

| NC | *pKMT2D* | NC | *pKMT2D* | NC | *pKMT2D* | Marker |
| --- | --- | --- | --- | --- | --- | --- |

NC: negative control, *pKMT2D*: plasmid encoding *KMT2D*.

| 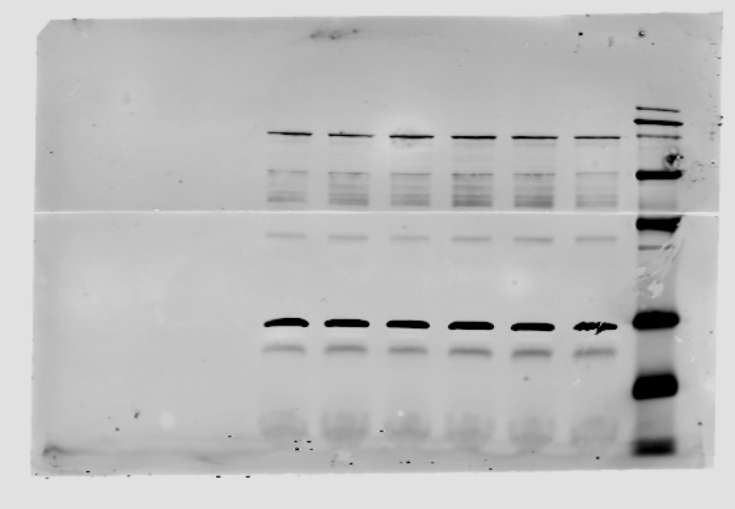 | **HSP90** |
| --- | --- |
|  | **H3K4** |
| 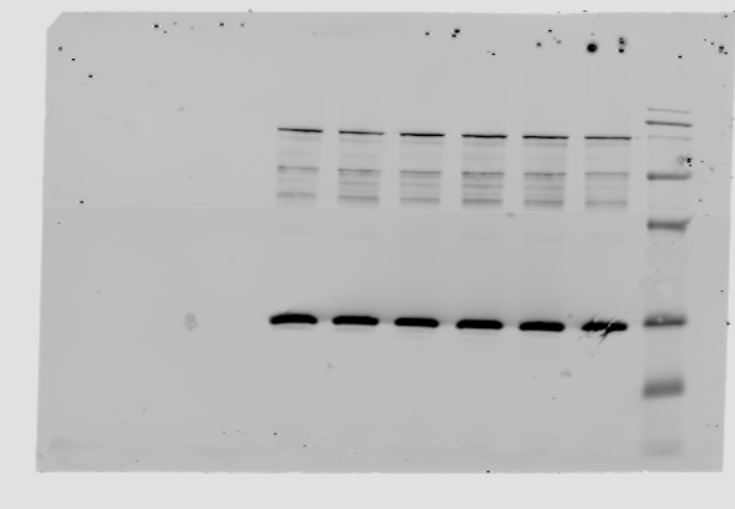 | **H3** |

**IM-R2**

| NC | *pKMT2D* | NC | *pKMT2D* | NC | *pKMT2D* | Marker | 7 unrelated samples |
| --- | --- | --- | --- | --- | --- | --- | --- |

NC: negative control, *pKMT2D*: plasmid encoding *KMT2D*.

| 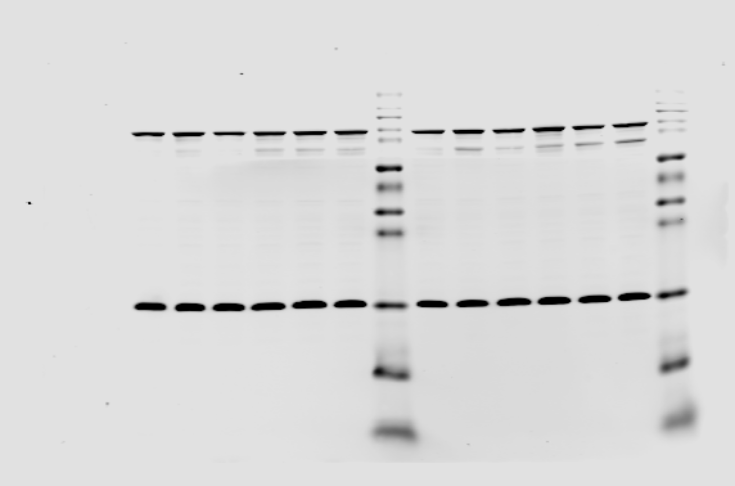 | **HSP90** |
| --- | --- |
|  | **H3K4** |
| 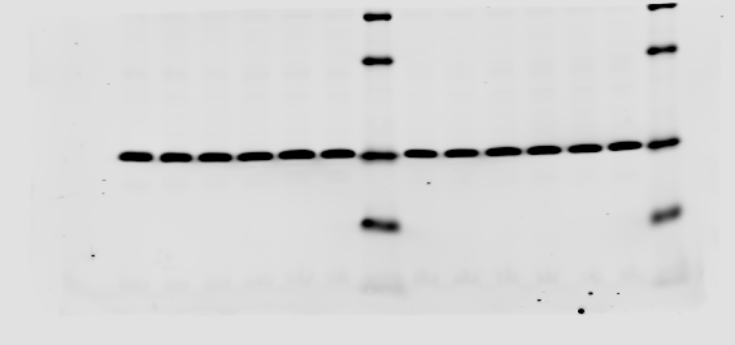 | **H3** |

**Fig. 4: *KMT2D* p.(Arg191Trp) impairs the response to imatinib.** N = 3.

| NC | *pKMT2D*  WT | *pKMT2D*  p.(R191W*)* | NC | *pKMT2D*  WT | *pKMT2D*  p.(R191W*)* | NC | *pKMT2D*  WT | *pKMT2D*  p.(R191W*)* | Marker |
| --- | --- | --- | --- | --- | --- | --- | --- | --- | --- |

NC: negative control, *pKMT2D*: plasmid encoding KMT2D, WT: wild-type.

| 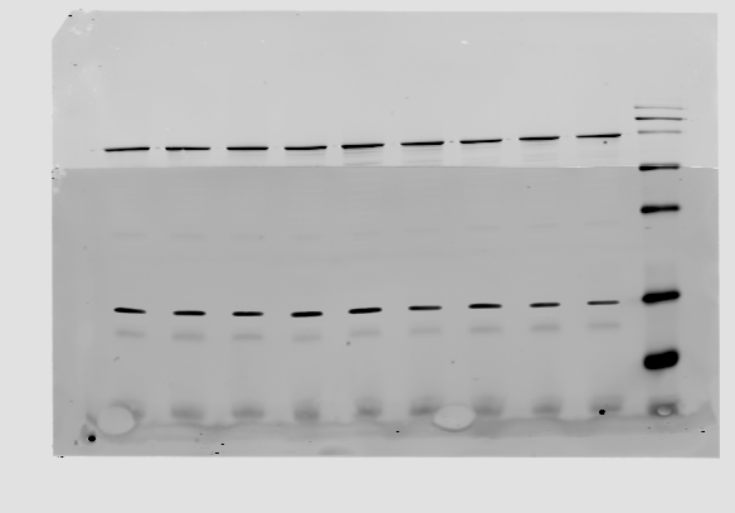 | **HSP90** |
| --- | --- |
|  | **H3K4** |
| 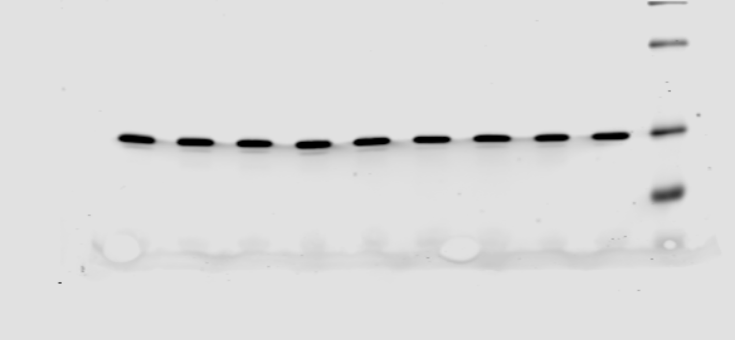 | **H3** |

**Fig. 5: *KMT2D* p.(Arg191Trp) impairs the response to imatinib.** N = 3.

| Marker | *pKMT2D*  p.(R191W*)* | | *pKMT2D*  WT | | NC | |
| --- | --- | --- | --- | --- | --- | --- |
|  | NTC | LSD1 inhibitor | NTC | LSD1 inhibitor | NTC | LSD1 inhibitor |

NC: negative control; NTC: no treatment control; WT: wild-type

| 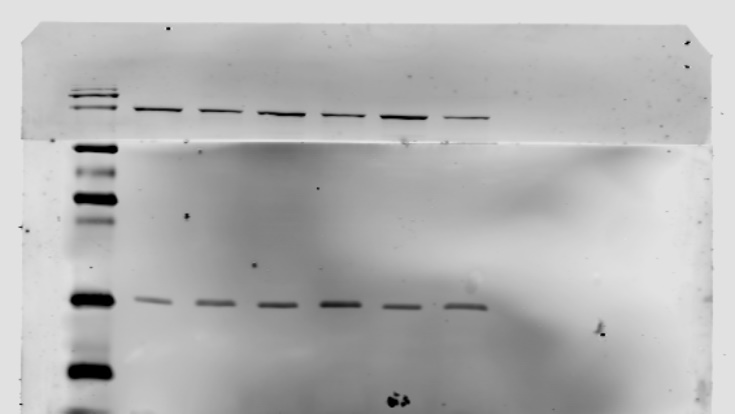 | **HSP90** |
| --- | --- |
|  | **H3K4** |
| 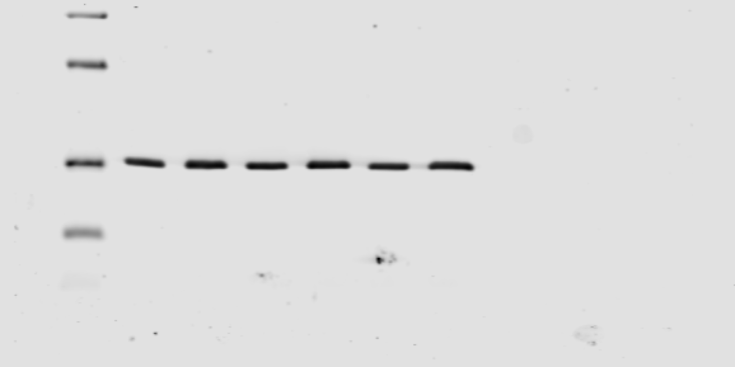 | **H3** |

| Marker | *pKMT2D*  p.(R191W*)* | | *pKMT2D*  WT | | NC | | Marker | *pKMT2D*  p.(R191W*)* | | *pKMT2D*  WT | | NC | |
| --- | --- | --- | --- | --- | --- | --- | --- | --- | --- | --- | --- | --- | --- |
|  | NTC | LSD1 inhibitor | NTC | LSD1 inhibitor | NTC | LSD1 inhibitor |  | NTC | LSD1 inhibitor | NTC | LSD1 inhibitor | NTC | LSD1 inhibitor |


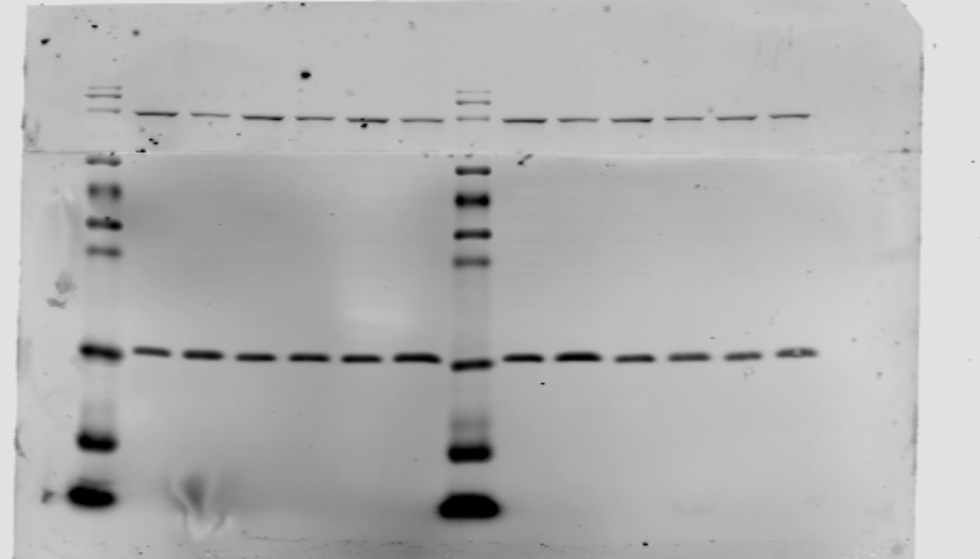

Supplement: Supplementary file 1 [file DataSheet1.docx]
