# Supplementary material for: The role of the lysine histone methylase KMT2D in chronic myeloid leukemia
Source: Front Pharmacol. 2025 Sep 16;16:1652373. doi: 10.3389/fphar.2025.1652373 (PMC12481891; doi:10.3389/fphar.2025.1652373)

Supplementary Figures

Supplementary Fig. S1. *KMT2D* expression in TKI-resistant cell lines. *KMT2D* mRNA expression was measured using RT-qPCR in treatment-naïve and TKI-resistant K-562 cells (resistant against 2 µM imatinib) harboring *KMT2D* wild-type. Expression was analyzed in three biological replicates of imatinib resistance. Data were normalized to *TBP*, *GAPDH* & *18 S* and treatment-naïve cells. Statistical analyses were performed using One-way ANOVA followed by Dunnett's test or student's t-test. N = 3. Error bars indicate standard deviation. ***: p < 0.001.

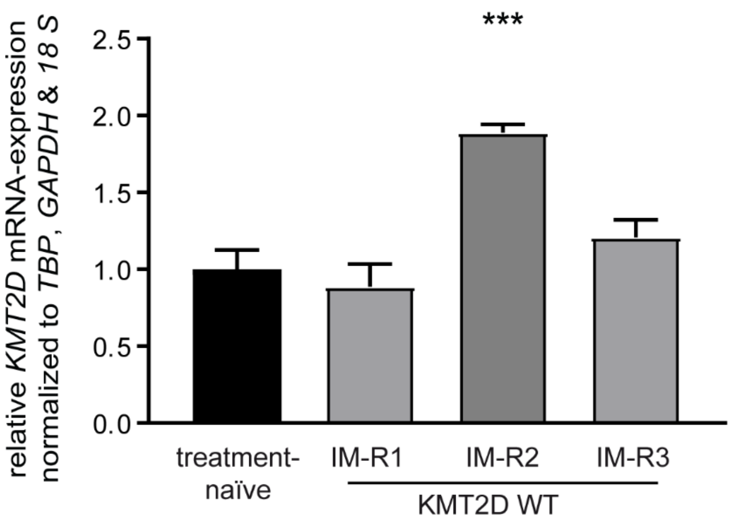

Supplement: Supplementary file 2 [file Image1.pdf]
